# The effects of classroom acoustic quality on student perception and wellbeing: a systematic review across educational levels

**DOI:** 10.3389/fpsyg.2025.1586997

**Published:** 2025-08-11

**Authors:** Alice Mercugliano, Arianna Corbani, Lucia Bigozzi, Giulia Vettori, Oriana Incognito

**Affiliations:** ^1^Department of Education, Languages, Intercultures, Literatures and Psychology, University of Florence, Florence, Italy; ^2^Department of Human Sciences, Università Telematica degli Studi IUL, Florence, Italy

**Keywords:** acoustic quality, school environment, wellbeing, reverberation, students' perception

## Abstract

The acoustic quality of the school environment is crucial in enhancing learning and contributing to classroom wellbeing. The aim of this systematic review was 2-fold: (1) to investigate students' perspectives on listening in different learning contexts, with a focus on noise and reverberation in the classroom; and (2) to investigate the impact of indoor acoustic quality and reverberation on wellbeing. Following the preferred reporting items for systematic reviews and meta-analyses (PRISMA) procedures to identify peer-reviewed studies, 12 studies met the inclusion criteria. Methodological quality was assessed using an existing framework. The results highlight an age-related progression in acoustic awareness from preschool children with limited differentiation to older students with refined perceptions. The impact of acoustics on wellbeing extends beyond learning to social relationships, motivation, and engagement, with older students exhibiting greater sensitivity than younger students. A research gap exists in preschool settings owing to methodological challenges in assessing young children's experiences. This review highlights the need for improved acoustic standards, tailored interventions, and awareness programmes to improve learning environments and wellbeing.

## 1 Introduction

Learning in a school environment requires a continuous interaction between cognitive and environmental processes. Since learning in school depends primarily on verbal communication, it is essential that the sound environment promotes a clear understanding of what teachers and peers say. Here, it is important to distinguish between sound, which is defined as any auditory stimulus including speech, and noise, which refers to undesirable or disruptive sound that interferes with listening (European Environment Agency, 2010). Acoustic conditions in classrooms can significantly impact children's academic abilities, with direct implications for educational success (Fernandes et al., 2019; Dockrell and Shield, 2006). Adverse acoustic conditions significantly impact cognition and learning Pellegatti et al., (2023). Particularly for school-age children, these effects can affect the long-term development of cognitive skills, which are crucial for academic performance and future opportunities (Clark and Paunovic, 2018). (Pellegatti et al. 2023) noted that an inadequate acoustic environment impairs basic cognitive functions, such as attention and memory, which are essential for learning and academic success (Gathercole et al., 2004; Stevens and Bavelier, 2012). However, exposure to noise has a negative impact on the performance of complex cognitive tasks, such as reading, writing, arithmetic, and numeracy, which are essential for everyday school activities (Papanikolaou et al., 2015; Dockrell and Shield, 2012).

## 2 Literature review

Recent systematic reviews have highlighted the crucial role of classroom acoustics in students' learning and wellbeing (Mealings and Buchholz, 2024; Gheller et al., 2024), with specific reference to the negative effects of noise on these factors. For instance, Gheller et al. (2024) emphasized that both speech, such as classroom activity noise or multitalker babble, and non-speech, such as road traffic, noises negatively affect children's academic performance, particularly their verbal working memory and reading ability. These findings highlight the importance of classroom acoustics as a critical factor in supporting students' learning and wellbeing. The review by Mealings and Buchholz (2024) also suggested that exposure to noise, which is often categorized as either chronic or acute, negatively affects children's cognitive abilities, including attention and memory. Similarly, recent meta-analyses by Fretes and Palau (2025) and Fernández-Quezada et al. (2025) confirmed that exposure to noise, primarily from external sources such as road, rail and air traffic, as well as some internal school noise, negatively affects cognitive performance in children and adolescents, particularly in terms of memory, attention and reading skills.

Previous studies have shown how acoustic quality also affects children's working memory, language comprehension, and attention (e.g., Dockrell and Shield, 2006; Connolly et al., 2019; Mealings, 2022). These cognitive functions are particularly sensitive to auditory distractions. Excessive or inappropriate noise can overload working memory, reduce attentional resources, and impair the processing of verbal information. For instance, children may require more cognitive effort to filter out background noise, which leaves fewer resources available for learning tasks (Mealings, 2022; Vettori et al., 2022).

Research on the effects of noise and reverberation in school contexts has mainly focused on performance in academic tasks (e.g., reading, mathematics, and memory). Both acute and chronic noise exposure are key factors affecting children's cognitive performance and listening skills. Acute noise refers to short-term exposure to noisy or disturbing sounds, whereas chronic noise refers to long-term exposure to environmental noise. Acute noise, such as sudden interruptions, impairs speech perception, listening comprehension, and short-term memory, whereas chronic noise, particularly in classrooms with high reverberation, is associated with poor performance on verbal tasks and reduced reading ability (Klatte et al., 2010b, 2013). These effects are more pronounced in children than in adults, as younger learners' executive functions are still developing (Zelazo et al., 2004) and a limited ability to compensate for noisy environments (Connolly et al., 2013).

The acoustic quality in schools is influenced by several factors, including outdoor noise, i.e., sound from the hallway or adjacent classroom, and indoor classroom noise, which is generated mainly by interactions between students and teachers during teaching activities. A fundamental aspect of indoor acoustics is reverberation, a phenomenon that occurs when sound bounces off surrounding surfaces, creating multiple sound reflections. In their review, Shield and Dockrell (2003) reported the results of Bradley's (1986) analysis of classroom acoustic conditions and speech intelligibility. Bradley concluded that an appropriate background noise level was 30 dB(A), and that optimum reverberation times should range between 0.4 and 0.5 s to support effective communication in learning environments. These values are consistent with the recommendations set out in the international standard ISO 3382-2 (International Organization for Standardization, 2008), which defines the measurement methods and acceptable limits for the reverberation time of ordinary rooms, including classrooms. In a classroom, reverberation amplifies both sound and noise, creating echoes and interference that make it difficult to understand voices, and interferes with educational activities. A high reverberation time negatively affects speech recognition, working memory, and verbal memory in children, impairing processes essential for academic success (Vettori et al., 2022). Peng et al. (2015) found that good acoustic quality in classrooms improved both learning outcomes and students' wellbeing at school. After acoustic treatment in their classroom, primary school children reported lower levels of noise and reverberation, such as noise from the hallway, outdoor traffic, and adjacent classrooms, as well as decreased perceived indoor reverberation. This indicates that it helped them to concentrate more easily and hear the teacher more clearly.

### 2.1 Literature gaps

Although noise and reverberation can affect cognitive performance and listening skills (e.g., Klatte et al., 2010b, 2013), few studies have considered students' metacognitive perceptions of these environmental factors (McFarland and Dealtry, 2017). Metacognitive perception of noise refers to individuals' awareness of listening conditions and their impact on their ability to learn, concentrate, and interact with others. This approach is essential to understand how students perceive and are aware of noisy conditions and their impact on their wellbeing and performance. In a study published in Massonnié's (2020) doctoral dissertation, the author showed that children (aged from 8 to 11 years old) with greater sound awareness who participated in mindfulness interventions designed with teachers reported reduced feelings of noise interference and annoyance and an increased sense of wellbeing. Moreover, regarding the impact on performance, perceiving poor acoustic quality leads to increased cognitive effort, which is associated with greater difficulty in processing and maintaining information in the short and long terms (Battagliarin et al., 2024). For instance, in a systematic review, Mogas-Recalde et al. (2021) observed how the perception of noise in the classroom affects students' abilities and behaviors, which vary according to age, sex, and other individual variables (such as atypical developmental conditions). This systematic review examined the effects on students and teachers, with only a partial focus on students. Therefore, another limitation of the current literature concerns participants, who are generally teachers or primary school children; the Empirical knowledge on adolescents and preschool children thus remains limited. This limitation is particularly relevant in the case of preschool children, for whom the concept of metacognition may be difficult to understand due to developmental constraints. However, several studies have shown that children aged 3–5 can exhibit early forms of metacognitive awareness. For instance, they may offer opinions on their own thought processes or listening experiences, demonstrate an awareness of their mental states, or spontaneously refer to mental processes without being prompted (Gopnik and Slaughter, 1991; Louca-Papaleontiou et al., 2012; Gonzales, 2015). Furthermore, studies on the development of theory of mind, such as that by Bigozzi et al. (2016), suggest that children are able to distinguish between appearance and reality and reflect on their own and others' mental content by the ages of 3 years old. This demonstrates early forms of metacognitive awareness. These findings challenge the idea that preschoolers lack metacognitive abilities altogether, supporting the inclusion of this age group in investigations of subjective experiences related to acoustic environments.

Moreover, studies like that by Dockrell and Shield (2004) and Brännström et al. (2017) have shown that children (aged from 6 to 13 years old) can judge noise in their environment and that different aspects of noise must be considered. To our knowledge, however, the relationship between indoor classroom noise perception and students' perceived wellbeing has been under-researched.

Thus, this review aims to extend the existing knowledge on the influence of acoustic conditions on performance and general wellbeing by considering a wider variety of participants and conditions.

## 3 This study

A systematic review in this field is needed to fill the gaps in the research on students' metacognitive perceptions of acoustic conditions in school environments. Although the existing literature has investigated the impact of factors such as noise and reverberation on children's cognitive performance, few studies have focused on how students perceive and respond to these conditions. This gap is critical because how students subjectively experience noise and reverberation can provide useful insights into their cognitive and emotional wellbeing, as well as their ability to engage and learn effectively in different acoustic environments.

Focusing on metacognitive perceptions allows researchers to explore how students are aware of and adapt to challenging listening conditions, including strategies for overcoming distractions and mitigating the negative effects of poor acoustics. Such perceptions are influenced by the objective acoustic characteristics of the environment and by individual differences, including age. Therefore, identifying the differences in perception between different levels of education, from kindergarten to secondary school, will help define practical recommendations for adapting to school environments according to the age and cognitive needs of students.

In this review, wellbeing is considered a multidimensional construct encompassing emotional (e.g., discomfort, annoyance, frustration), cognitive (e.g., poor concentration, mental fatigue), and school-related (e.g., motivation, perceived classroom climate, satisfaction with learning experiences) aspects. While definitions and indicators may differ between studies, our aim is to capture students' perceived wellbeing in relation to their acoustic experiences in the classroom.

To address these research gaps, two aims were defined:

1) To investigate students' perspectives and awareness related to listening in various learning situations, with a specific focus on indoor classroom noise and reverberation across different educational stages (from preschool to secondary school), taking into account developmental differences in metacognitive abilities.
2) To examine how the perceived quality of indoor acoustic environments affects students' wellbeing, considering emotional, cognitive and school-related dimensions, and how such effects may be mediated by students' ability to reflect on and interpret their acoustic experiences.

## 4 Methods

The preferred reporting items for systematic reviews and meta-analyses (PRISMA) guidelines were applied to guide the methodology and report the results (Page et al., 2021).

### 4.1 Data sources and search strategy

A comprehensive search of the PsycInfo and Education Source (via EbscoHost), Scopus, and Embase databases was performed in July 2024. A combination of keywords with AND and/or OR Boolean operators regarding parameters such as student age, level of education, school environment, reverberation, indoor acoustics, and metacognition were used.

The search strategies developed for each database are presented in Appendices A–C.

In addition to the database searches, a targeted integrative search was conducted to ensure the comprehensiveness of the review. This included searches in specific peer-reviewed journals relevant to the topic, such as the Journal of the Acoustical Society of America, Australasian Journal of Early Childhood, and targeted searches for works by key authors recognized as experts in the field, such as McFarland and Visentin.

### 4.2. Study selection and eligibility criteria

The Rayyan app dedicated to systematic reviews was used (Ouzzani et al., 2016). After independently screening the abstracts and titles, two reviewers (A.C. and A.M.) included all the pertinent papers in the full-text review to verify their eligibility. In cases of disagreement, they reached a unanimous decision by discussing the justifications for including or excluding articles. The inclusion and exclusion criteria were established as follows:

#### 4.2.1 Inclusion criteria

1. Empirical studies exploring student's perception and awareness of the impact of indoor classroom acoustic conditions (e.g., reverberation, background noise) on their learning environments, listening conditions, attention, emotional wellbeing, or perceived comfort or annoyance.
2. Studies involving children and adolescents enrolled in preschool, primary school, and/or secondary school.
3. Studies employing quantitative, qualitative, or mixed-methods designs.
4. Use of self-report methods directly involving students (e.g., surveys, questionnaires, interviews, focus groups).
5. Peer-reviewed journal articles, with no restrictions were applied regarding publication date or language.

#### 4.2.2 Exclusion criteria

1. Studies investigating exclusively student perceptions and awareness of the impact of outdoor noise levels on their learning environments, concentration, emotional wellbeing, or comfort, as indoor and outdoor noise are two distinct sources of discomfort that need to be addressed separately. This is important for acoustic management and intervention strategies.
2. Studies focusing exclusively on the effects of classroom acoustic quality on children's academic performance and cognitive tasks, without reference to student perception.
3. Studies focusing on different target populations (e.g., teachers, parents, school staff, and children with hearing problems).
4. Gray literature (book chapters, doctoral dissertation, theoretical review, commentaries).
5. Non-availability of the full text.

### 4.3 Data collection and quality assessment

Two evaluators extracted the data from the included studies. The reviewers reached consensus to settle any disagreements. For each independent study sample, information was extracted from the publication (e.g., authors, year), study design, country, aim, sample characteristics (e.g., sample size and age), type of educational institution, measure used, and results obtained.

The methodological quality of the included studies was evaluated using a tool proposed by Hjetland et al. (2017) for observational studies. The following quality indicators were analyzed for each study: sampling, selection, instrument quality, test reliability, floor or ceiling effects, attrition, missing data, latent variables, and statistical power/sample size. A score of 0, 1, or 2 was chosen for each indicator; a score of 0 indicated a low risk of bias, whereas a score of 1 or 2, depending on the indicator, indicated a high risk of bias. There was also a greater risk of non-reporting. The overall score for each study (ranging from 0 to 11) was calculated by adding the values. After discussing the potential interpretations of the instrument used, two evaluators (A. C. and A. M.) separately ranked each study with a 98% inter-rater agreement.

## 5 Results

From the initial 4,845 records retrieved through literature searches, after removing duplicates, 4,393 studies remained and were screened for titles and abstracts by two independent evaluators (A. C. and A. M.). The screening phase was first applied to titles and abstracts, and subsequently to full texts. This process resulted in the identification of 96 candidate studies, which subsequently underwent review for potential inclusion in the systematic review, and 87 were excluded as they did not meet the inclusion criteria; the number of articles and the reasons for exclusion are described below.

**Figure 1:**
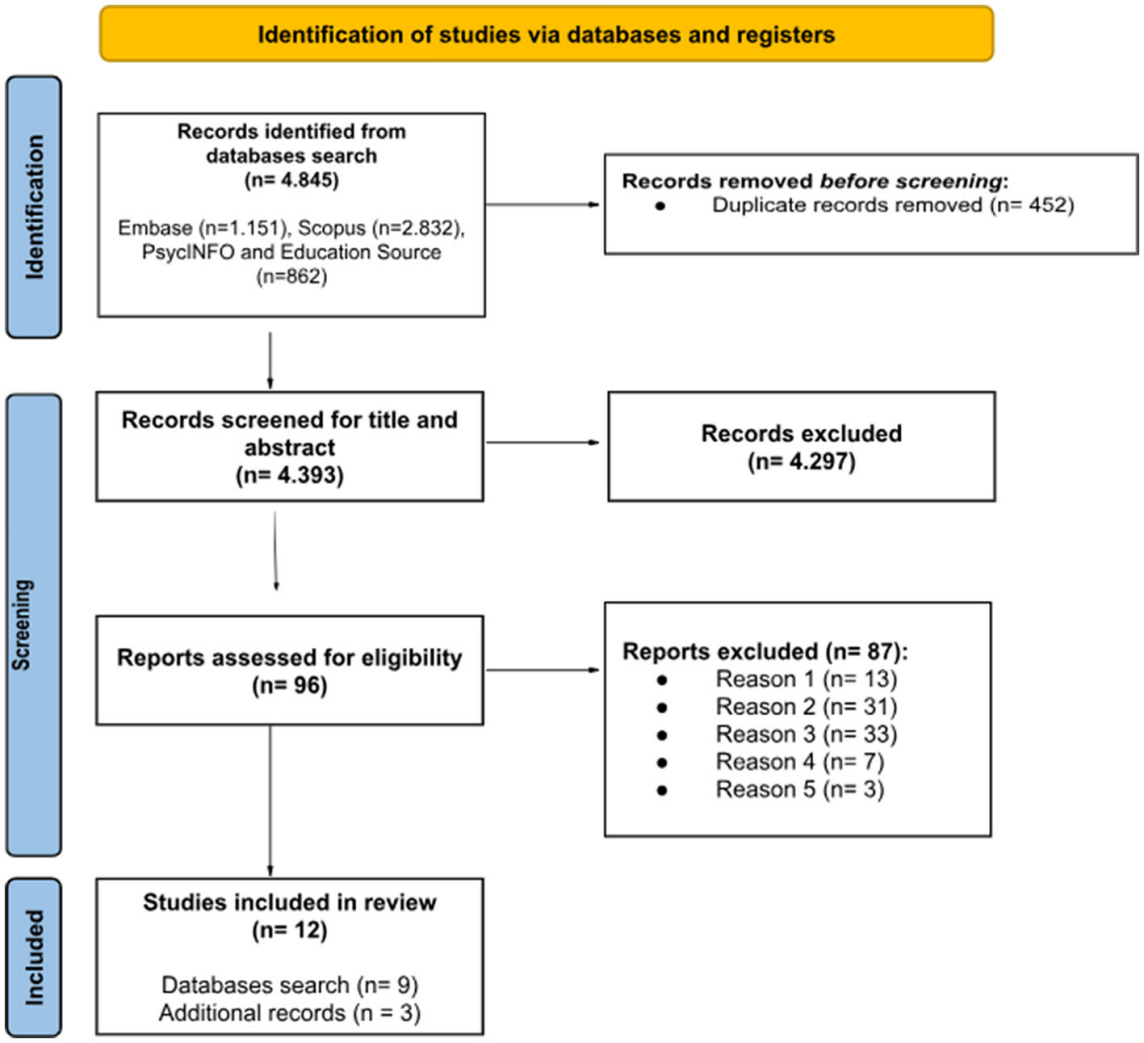
PRISMA flow diagram for new systematic review (Page et al., 2021).

Finally, 12 studies were selected, specifically nine studies from a database search and three studies from a targeted integrative search conducted in January 2025. In Figure 1, we report the PRISMA scheme, detailing the selection process adapted from Page et al. (2021).

### 5.1 Quality assessment

According to the data gathered, examination of the included papers indicated possible bias concerns across a range of research variables. The results of each quality indicator are briefly described below.

- Sampling: Of the 12 studies analyzed, two demonstrated robust sampling methods, scoring 0, while 10 showed methodological concerns. Additionally, we coded whether the sample in each study was selected (*N* = 5) if a set of criteria guided the selection process. This finding suggests sampling limitations (convenience) in the reviewed studies.
- Instrument quality: Most studies have raised concerns regarding instrument quality. Two studies showed significant limitations, seven demonstrated moderate limitations, and three demonstrated strong instrument quality.
- Test reliability: Test reliability is generally well-addressed across studies. Nine studies demonstrated good reliability, while only three studies showed reliability concerns.
- Floor/ceiling effect: None of the studies reported floor or ceiling effects, indicating consistent methodological limitations.
- Attrition: Most studies showed strong retention rates, whereas two studies demonstrated attrition concerns.
- Missing data analysis: The studies handled missing data appropriately, whereas three studies showed limitations in their missing data approach.
- Latent variables: Seven studies demonstrated appropriate handling of variables, while five studies showed some concerns.
- Statistical power: Nine studies demonstrated adequate statistical power (more than > 70 participants), whereas three studies showed significant limitations in their sample size (fewer than 70 participants).

**Figure 2:**
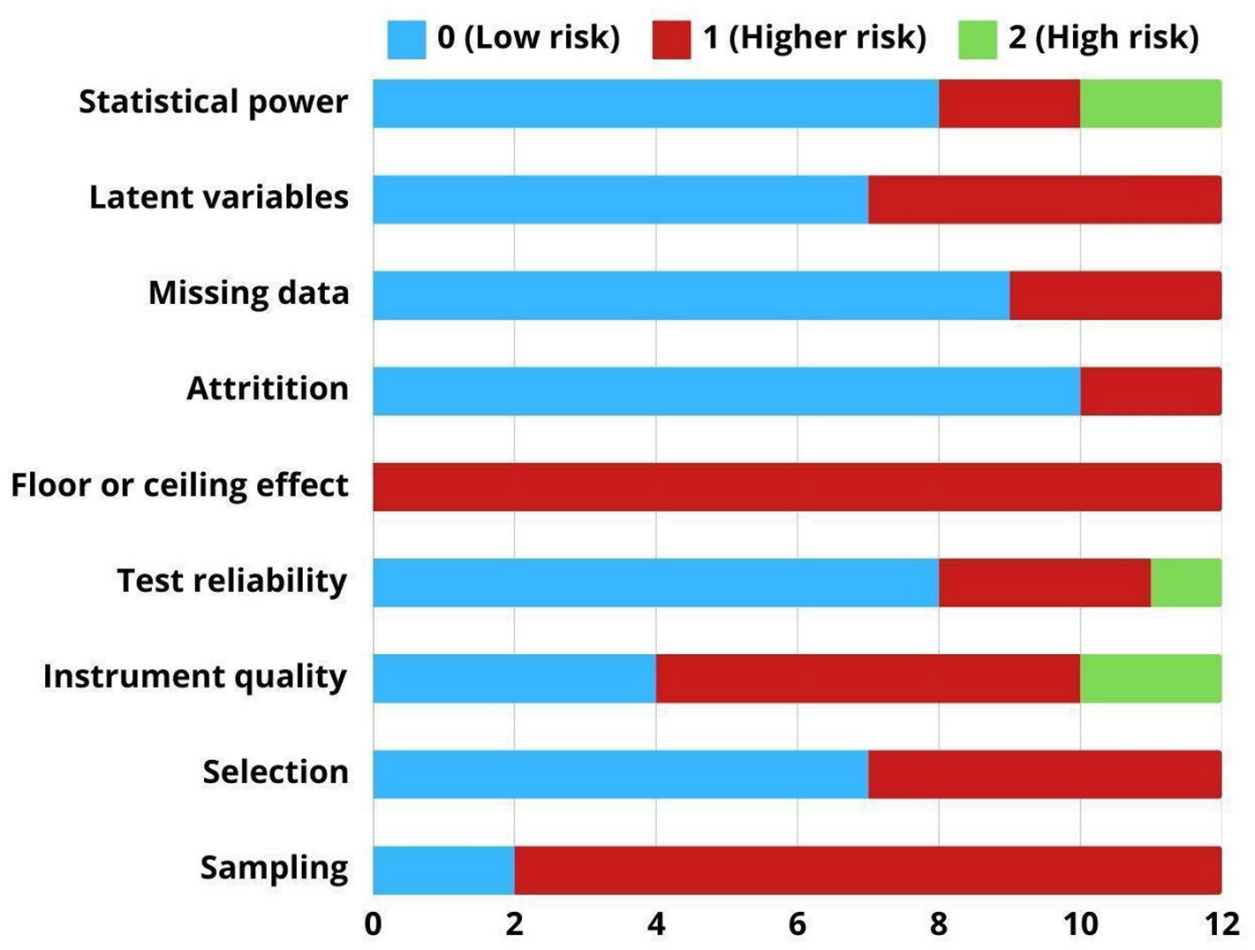
Risk of bias across included studies according to predefined quality domains.

Figure 2 shows a thorough evaluation of the quality of the studies. This highlights the potential risk of bias across various studies based on the identified issues. Some indicators had three possible values (0, 1, and 2), whereas others had only two (0 and 1). Appendix D contains the specific individual scores for each study.

Studies with the lowest total risk of bias scores were Dockrell and Shield (2004), Klatte et al. (2010b), Lundquist et al. (2003), and Vettori et al. (2024), each obtaining a total score of 3. These studies demonstrated strengths in test reliability, appropriate handling of latent variables, and statistical power, with some also reporting more robust sampling procedures. By contrast, studies such as McFarland and Dealtry (2017) (score = 9) and Skarlatos and Manatakis (2003) (score = 8) presented higher risk of bias, particularly due to small sample sizes, weak reliability reporting, or unclear sampling methods.

A detailed description of each study's score by criterion is reported in Appendix D.

### 5.2 Characteristics of the studies

The results will be presented in a narrative mode based on what the study authors reported, and there will be no attempt to aggregate the data in a meta-analysis. Appendix E provides an overview of the features of each study.

These studies were conducted across various countries, including Italy, Greece, Turkey, the United Kingdom, Germany, Sweden, Finland, Australia, and Corsica and highlighted a diverse range of educational contexts. The sample sizes varied significantly across studies, ranging from 50 participants in smaller experimental or qualitative studies to over 2,000 participants in large-scale surveys.

The age groups of the participants ranged from preschool and kindergarten children (3–5-years-old) to high school students (up to 20-years-old). In most studies (*n* = 9), participants were primary school children, typically aged 6–12 years (e.g., Klatte et al., 2010b; Vettori et al., 2024). Two studies included older students ranging from upper elementary grades to high school (aged 12–20 years; Lundquist et al., 2003; Skarlatos and Manatakis, 2003). Only one study included preschool and kindergarten children aged 3–6 years (McFarland and Dealtry, 2017).

A comprehensive review of the included studies revealed a complex interplay among acoustic conditions, student experiences and perceptions, and wellbeing outcomes across educational levels. The results will be discussed based on and divided by the proposed objectives in the review, paying particular attention to the variation in results according to the age of the students involved and considering different listening contexts and situations.

Specifically, nine studies investigated students' perceptions of listening in various learning situations (answering the first aim of the review), eight studies assessed the impact of acoustic quality and indoor reverberation on wellbeing (answering the second aim of the review), and only five studies investigated both aspects in depth, satisfying both aims of the review.

Table 1 provides a concise summary of the 12 studies included in the review, including their objectives, methods, and key findings. A more detailed description of each study is available in Appendix E.

**Table 1:** Summary of the 12 studies included in the review.

**References**	**Sample characteristic**	**Aim**	**Method and instrument**	**Result**
1. Astolfi et al. (2019)	340 primary school children, age 6–8	Investigate the impact of classroom acoustics on students' perceived wellbeing and noise disturbance	Acoustic measures; Self-report questionnaires on wellbeing and noise disturbance	Long reverberation times are associated with lower enjoyment and higher noise disturbance. Good acoustics is more strongly associated with positive wellbeing scores than poor acoustics.
2. Bulunuz (2014)	611 primary school children, age 7–13	Examine students' perceptions of noise pollution and compare state and privately funded school settings	Noise level measurements; *Ad-hoc* questionnaire on noise perception	Agreement between subjective reports and acoustic data confirms perception accuracy. Noise annoyance is higher in state funded schools than in privately funded.
3. Dockrell and Shield (2004)	2,036 primary school children, age 6–7 and 10–11	Investigate how children perceive different classroom noise sources and their impact on hearing the teacher	Questionnaire on environmental noise sources; Smiley-face rating scale	Children distinguish between noise situations affecting hearing (e.g., noise inside/outside the classroom, or doing a test). Younger children report more difficulty overall.
4. Klatte et al. (2010a)	487 primary school children, age 7–8	Examine the impact of classroom reverberation time on students' wellbeing	Acoustic measurements; Questionnaires on noise annoyance, and emotional-social school experience	Longer reverberation times are associated with higher noise annoyance and lower scores on achievement motivation, teacher relationships, classroom climate, and social integration.
5. Lundquist et al. (2003)	443 secondary school children, age 12–15	Assess the relationship between classroom noise levels and students' mood and attentional states	Sound level meter and digital recorder; Mood questionnaire	No significant correlation is found between noise levels and background noise or number of students. Higher sound levels do not predict increased annoyance or inattention.
6. Massonnié et al. (2022).	112 primary school children, age 8–11	Explore how attentional and cognitive factors influence children's perception of noise	Children's reactions to noise questionnaire; Attentional Control Switching Scale; Mind Wandering Questionnaire	Five factors emerge: perceived noise, attentional capture, hearing difficulties, interference, and annoyance. Mind wandering increased interference and annoyance.
7. McFarland and Dealtry (2017)	69 preschool children, age 3–5	Explore preschool children's perceptions of hearing experiences in different classroom situations	Self-administered booklet using verbal, affective, and visual/arts-based responses	Children report better hearing at the front of the mat and during quiet activities. More difficulty is reported during peer noise and when seated at the back.
8. Papanikolaou et al. (2013)	594 primary school children, grade 5–6	Investigate children's perception of internal and external classroom noise and related distress	*Ad-hoc* questionnaire on noise sources and perceived annoyance	Internal sources are classmates and corridor noise. Annoyance levels vary by city and gender, with girls reporting greater impact on academic performance.
9. Pirilä et al. (2020)	50 primary school children, age 8–9 and 10–11	Evaluate changes in students' perception of classroom noise and teacher audibility after acoustic interventions	Acoustic measurements; Questionnaires on annoying noises and teacher voice perception	Post-intervention, older students report improved voice clarity and reduced listening effort. Both age groups note less annoyance from furniture and corridor noise; one group also from peers talking.
10. Skarlatos and Manatakis (2003)	411 secondary students, age 12–20	Examine the relationship between classroom noise levels and perceived discomfort among students	Acoustic measurement; Questionnaire on noise perception	Older students reported greater discomfort due to noise. A moderate positive correlation was found between noise levels and perceived discomfort.
11. Vettori et al. (2024)	213 primary school children, age 6–11	Investigate children's perspectives on listening during learning under different acoustic conditions	Acoustic measurements; Questionnaire on listening perspectives	Significant interaction between acoustic condition and grade: the acoustic environment affects listening perception in specific grades (better in older students).
12. Visentin et al. (2023)	130 primary school children, age 8–10	Assess children's perception of actual and ideal classroom sound environments	Acoustic measurements; Questionnaire on subjective sound perception	Voices of students are the main noise source, mostly unpleasant. Increased noise decreases comfort. Younger children feel more comfortable than older ones.

### 5.3 Students' perceptions of listening in different acoustic conditions

Regarding students' perspectives on listening, research demonstrates age-specific patterns in how children perceive their listening under various acoustic conditions within daily classroom activities.

In early education settings, McFarland and Dealtry (2017) investigated the perspectives of 3-year-old children (*N* = 69) regarding the prevalence of listening difficulties and group activities, during which these challenges are most commonly reported. This study showed that preschoolers could effectively articulate their listening experiences when provided with appropriate assessment tools. Employing a participatory approach and a self-report instrument to gather children's perspectives on their listening experiences, this study revealed that preschoolers have difficulty listening when multiple sound sources compete for their attention, particularly during group activities or when they are physically distant from educators (e.g., group story reading sessions).

As children move to primary school, their perceptions of acoustic conditions become more articulated. Studies by Astolfi et al. (2019) and Vettori et al. (2024), which focused on early primary school children, found that although younger children were less able than older peers to discriminate between different acoustic conditions, such as varying levels of background noise and reverberation, yet still experienced increased cognitive effort in poor acoustic environments. This is particularly relevant during group activities or in the presence of background noise.

In the study by Astolfi et al. (2019), first-grade children (aged 6–8 years; *N* = 340) completed two questionnaires to evaluate their subjective perception of wellbeing and noise disturbance while in the classroom. The results showed that the children reported higher disturbance during silent tasks and higher intensity and disturbance during group activities, as reflected by higher mean scores on the respective rating scales. Noise measurements were performed under both conditions to represent typical classroom scenarios. Both conditions were ensured by the teacher asking the children to be silent and to speak as in a traditional group lesson. The results confirmed that poor classroom acoustics, especially high reverberation times, worsen listening conditions. Consequently, increased reverberation levels in indoor environments increase the perceived noise and disruptive effects on children. Whereas, Astolfi et al. (2019) highlighted the role of reverberation in students' perceived disruption, Vettori et al. (2024) focused on the effects of improved acoustics on speech comprehension in the classroom. Vettori et al. (2024) investigated the effect of reverberation time on the perception of teachers' and peers' voices during daily school activities among primary schoolchildren (aged 6–11 years; *N* = 213). To this end, a questionnaire was administered to assess the children's perception of voice clarity during classroom activities (e.g., items from the questionnaire: ‘When your teacher is talking and moving around the class, how well do you hear his/her voice?'). Comparing classrooms with poor acoustic quality, characterized by a long reverberation time, with classrooms with reduced reverberation time and equipped with sound-absorbing systems (i.e., good acoustic quality), children aged between 9 and 10 years reported more benefits from classrooms with better acoustic conditions. In contrast, younger children (aged 6–8 years) were less able to discriminate between different acoustic conditions. In particular, classrooms with longer reverberation times negatively impacted daily school activities, including group tasks, background noise, and the ability to distinguish between teachers' and peers' voices in noisy environments. In the same way, Pirilä et al. (2020) assessed the perception of annoying sound during daily school activities among primary school children aged 8–12 years (*N* = 50). Older children were significantly more negatively affected by activity noise than younger students [*F*_(1, 48)_ = 799,906, *p* = 0.01], although the reverberation time and background noise level detected in their classrooms were similar.

Further evidence of primary school children's noise perception was obtained from five other studies. Klatte et al. (2010b) highlighted that first and second grade students (aged 7–8 years; *N* = 89 and *N* = 398, respectively) could distinguish between different acoustic conditions (e.g., classroom with different reverberation time). In particular, indoor noise ratings were lower for children whose classrooms had short reverberations than for children from classrooms with medium and long reverberations (p < 0.001 in both cases). Similarly, Dockrell and Shield (2004) assessed primary school children's ability, from second to sixth grades (aged 6–11 years; *N* = 2,036), to discern different noise sources and their capacity to differentiate between good and poor listening situations in classrooms. Children reported awareness of various noise sources and classroom noise (e.g., sounds from classmates, group work, and teaching activities) that negatively impacted their ability—particularly among younger children—to hear the teacher, especially in noisy situations.

In addition to the aforementioned studies, Visentin et al. (2023) investigated sound perception in children aged 8–10 years (*N* = 130). Considering indoor sound sources, primary school children described both sounds generated inside the classroom, such as children's chatting, furniture scraping, and objects being moved or dropped, and sounds outside the classroom, such as children's or teachers' voices from adjacent classrooms, chairs or desks scraping upstairs, and people talking and/or moving in corridors. The results show that most of the identified noises came from the children themselves and that the most annoying noises were the scraping of furniture in the classroom and from the classrooms above. While Visentin et al. (2023) focused on specific sounds perceived by children (e.g., chattering, furniture noise, and voices from neighboring classrooms), Bulunuz (2014) investigated the general level of noise pollution in schools, adding a comparison with objective data and institutional differences. Bulunuz's (2014) research on primary school students (aged 7–13 years; N = 611) in state funded and privately funded institutions revealed significant findings regarding children's acoustic perceptions. Using a self-report questionnaire methodology complemented by objective acoustic measurements, this study demonstrated that students could accurately perceive and report noise pollution levels in their educational environment. The consistency between the students' subjective perceptions and objective reverberation measurements validates their ability to recognize acoustic conditions despite institutional differences (state funded and privately funded school).

At a higher educational level, a study conducted by Skarlatos and Manatakis (2003) in Greek secondary schools revealed significant findings regarding classroom acoustics and student perceptions (aged 15 years; *N* = 411). Despite measurements indicating consistently high reverberation times and significant background noise during both lessons and break periods, students' perceptions varied considerably. When asked to evaluate noise levels on a five-point Likert scale, 49% of the students rated classroom noise as unacceptable. Surprisingly, only 8% found break-time noise levels unacceptable, despite the decibel levels being higher than those of the classroom activities. Regarding speech intelligibility, 83% of the students consistently reported hearing their teachers clearly, while 13%, predominantly those seated at the back of the classroom, reported occasional difficulty in recognizing their teachers' words.

In conclusion, these studies reveal a significant progression in students' acoustic perceptions at different educational levels. While students across all age groups demonstrate the ability to perceive acoustic conditions accurately, as confirmed by the correspondence between subjective perceptions and objective measurements, their responses and awareness varies substantially with age. Notably, preschool children demonstrate a remarkable awareness of acoustic conditions and can identify and articulate listening difficulties, particularly during specific activities such as group storytelling or when physically distant from educators. This early acoustic awareness, though less discriminating than that in older students, challenges traditional assumptions about young children's perceptual capabilities. While most studies suggest that older students have increased sensitivity and ability to articulate their acoustic experiences despite being exposed to generally lower noise levels, some studies suggest more complex patterns, with factors such as habituation or cultural context potentially influencing perception (Johnson and Hannon, 2015). Reverberation is a critical factor amplifying the perception of disturbance, particularly during group activities.

### 5.4 Impact of acoustics conditions on student wellbeing

The impact of acoustic conditions on students' wellbeing has emerged as a significant variable across studies. Specifically, some studies conducted in primary schools (e.g., Astolfi et al., 2019; and Klatte et al., 2010b) have shown that poor acoustic conditions, particularly high reverberation times, negatively affect multiple aspects of student wellbeing. These effects extend beyond noise discomfort and affect social relationships, motivation, and the classroom's general atmosphere. Specifically, Astolfi et al. (2019) divided primary school students into “happy” and “unhappy” based on their scores on a scale of perceived wellbeing in terms of self-esteem, relationships with others, and enjoyment of school. Interestingly, they found that happy students were more sensitive to noise disturbances in classrooms with high noise levels or reverberations, suggesting a complex relationship between emotional wellbeing and acoustic perception. However, Klatte et al. (2010b) focused on the effects of short, medium, and long reverberation times on social and emotional experiences typical of school environments. Significant effects of reverberation were found for the factors “achievement motivation,” “relationship with teachers,” “classroom atmosphere,” and “social integration” (*p* < 0.01 for all reverberation times). Children in classrooms with long reverberation times evaluated these aspects less positively than children in classrooms with medium and short reverberation times (*p* < 0.05 in both cases).

Several studies have analyzed primary school children's perceptions of the school environment from a more general perspective, investigating both the awareness of unfavorable acoustic conditions and the ideal acoustic characteristics that children would like to have in their school environment. Using a questionnaire to collect student feedback on noise, Bulunuz (2014) observed the negative effects of noise on mental and physical health as well as its disruption of verbal communication and concentration. In privately funded schools, 69.3% of students perceived that noise pollution was present in their school, and 73.1% were bothered by it, lower than the 83.9% and 83% of students in public schools, respectively. Further, Visentin et al. (2023) explored the perspectives and preferences of primary school children regarding the soundscape of their school environment, independent of the actual environmental characteristics of their classrooms. Overall, children seemed to prefer nature-related sounds that promote peacefulness and rest during lessons, suggesting that a completely silent state, which is difficult to achieve in a learning context, does not correspond to the pupils' ideal soundscape.

Other studies conducted with primary and secondary school students (5th to 12th grades) showed a more sophisticated understanding of and responses to acoustic conditions. In a study of primary school children (aged 8–11 years; *N* = 112), Massonnié et al. (2022) defined five dimensions related to children's perception of and reactions to noise: perceived noise in the classroom, listening difficulties, attention-seeking, interference, and noise annoyance. Exploratory factor analysis showed that noise annoyance and interference with learning-related activities were related but distinct dimensions. Regarding children's reported hearing difficulties (e.g., I can hear the teacher well when she talks to me), these predict both noise-related interference and annoyance (β = 0.34; *p* = 0.01 and β = 0.31; *p* = 0.02).

Papanikolaou et al. (2013) provided an overview of the effects of indoor noise among higher-grade primary school students. Papanikolaou et al. (2013) explored perceived indoor noise discomfort, perceived annoyance, and the ability to focus during classes between primary 5th and 6th graders (*N* = 594) attending school in various cities in Greece. Children whose school buildings were in large cities were more exposed to internal noise, such as sounds coming from school corridors and neighboring classrooms, and reported more annoyance. In addition, the study provides some evidence of sex differences in the perception of annoyance (*F* = 3.86, df = 1.590, *p* < 0.05), indeed, girls reported more annoyance, which caused them to be distracted during lessons.

In a study conducted at the Swedish school level, known as Grundskola,^1^ Lundquist et al. (2003) investigated noise levels in classrooms in three different schools for upper primary school children (aged 12–15 years; *N* = 443) and how these levels were influenced by the type of education, number of pupils, and grade, as well as possible associations with annoyance. The results suggest that during lessons, noise levels were caused by student activities (more in language lessons than in mathematics), talking, and furniture scraping. However, the research does not support the evidence of a correlation between activity sound level and background sound level or variation in the number of children in each class or year group (*F* = 3.0, *p* > 0.05), and does not support the idea that higher sound levels should increase annoyance and inattention.

Extending the focus on older students, Skarlatos and Manatakis (2003) observed a significant age-related pattern in noise perception and its effects among secondary school students (*N* = 411, mean age = 15 years). The researchers found a positive correlation (*r* = 0.61, *p* < 0.01) between noise levels and student discomfort, which was influenced by three factors: time of day, type of activity, and student age. While overall noise levels decreased as students aged, they showed increased sensitivity to acoustic disturbances.

Collectively, these studies demonstrate a complex relationship between acoustic conditions and student wellbeing across educational levels. The impact of acoustic conditions extends well beyond mere discomfort, with significant effects on social, motivational, and classroom relationships. Particularly consistent across studies is the finding that reverberation time emerges as a critical factor influencing multiple dimensions of student wellbeing, from achievement motivation to social integration (i.e., Klatte et al., 2010b; Astolfi et al., 2019). As shown across the included studies (see Table 1), a consistent developmental pattern emerges in acoustic perception and their impact on wellbeing. Older students show greater sensitivity to acoustic conditions and their impact on their wellbeing despite experiencing lower noise levels overall (e.g., Skarlatos and Manatakis, 2003), suggesting an evolutionary dimension of acoustic perception that deserves further investigation.

## 6 Discussion

This systematic review reveals a complex relationship between acoustic quality perception and wellbeing in educational settings and addresses two main objectives. Regarding the first objective, studies have consistently demonstrated the age-related progression of acoustic perception. This developmental pattern extends from preschoolers, who can easily identify basic listening difficulties in different classroom situations, such as group activities, or when they are physically distant from teachers (McFarland and Dealtry, 2017), to primary school students, who can clearly distinguish between acoustic conditions, such as variations in reverberation time or background noise levels in the classroom (Astolfi et al., 2019; Vettori et al., 2024), and older students, who demonstrate a more sophisticated understanding of acoustic environments (Massonnié et al., 2022; Skarlatos and Manatakis, 2003). Therefore, at earlier developmental stages, particularly during preschool and early primary years, children demonstrate limited differentiation between varying acoustic conditions compared with older children. The developmental transition in acoustic perception may align with established theories of cognitive development, suggesting that younger children's reduced acoustic discrimination may result from their emerging metacognitive awareness and still-developing executive functions (Bablekou et al., 2023; Kuhn, 2000). As children grow older, their understanding of acoustic environments becomes more sophisticated: they are increasingly able to identify specific noise sources, assess the disruptive effects of different types of sound on their learning and concentration, and reflect on possible strategies to manage or reduce noise-related distractions.

Concerning the second objective, the relationship between acoustics and wellbeing was manifested in multiple dimensions. Different studies have highlighted how inadequate acoustic conditions affect immediate learning experiences, discomfort, and other specific aspects, including social relationships and peer interactions, academic motivation, emotional state, and school engagement (e.g., Klatte et al., 2010b; Visentin et al., 2023). For instance, Klatte et al. (2010b) found that noise exposure in schools significantly impairs verbal communication between peers, impairing cooperative learning and peer relationships. Similarly, Visentin et al. (2023) found increased levels of self-reported irritability and reduced emotional regulation in noisier classrooms, particularly during individual tasks. Massonnié et al. (2022) demonstrated that students exposed to better acoustic conditions showed greater academic engagement and concentration. These findings are usually derived from subjective measures, such as student self-reports and teacher assessments. This emphasizes the importance of perception in mediating the relationship between acoustic conditions and wellbeing outcomes. This multidimensional effect appears to evolve with age and exhibits a developmental pattern. Older students demonstrate heightened sensitivity to acoustic conditions and their effects on wellbeing despite generally experiencing lower noise levels in their learning environments (Skarlatos and Manatakis, 2003). This increased awareness may reflect more developed metacognitive abilities and a greater capacity to articulate the relationship between environmental conditions and personal wellbeing (Vrolijk et al., 2021). However, a significant research gap exists regarding acoustic impact on wellbeing in preschool settings. This lack of research might be attributed to two main factors: methodological challenges in assessing wellbeing in young children, particularly regarding their ability to self-report and articulate their experiences (Dockrell and Shield, 2006); and developmental considerations related to emerging metacognitive abilities, which may influence young children's capacity to recognize and express how acoustic conditions affect their wellbeing (Dockrell and Shield, 2006).

The studies generally focused on primary school children, with limited research on preschool or secondary school students, confirming the gap in the literature identified in this systematic review. The methodological approaches to data collection and analysis varied from quantitative measurements to qualitative assessments, with only a few studies, such as Bulunuz (2014) and Astolfi et al. (2019), effectively integrating objective acoustic parameters with subjective data. The results reveal the fundamental importance of considering both measures (e.g., Massonnié et al., 2022), as they capture distinct yet interrelated aspects of the acoustic environment. While physical acoustic parameters can be measured accurately with objective measures, their impact on learning and wellbeing must be understood through the personal experience and perception reported by students. The quality assessment of the included studies revealed varying levels of methodological rigor. While some studies have demonstrated robust designs and comprehensive reporting, others have shown limitations. Common methodological weaknesses include inadequate justification of sample size, limited control of environmental variables, and inconsistent reporting of acoustic measurement procedures. Additionally, several studies lacked clear descriptions of participant selection criteria or failed to account for potential confounding factors such as individual sensitivity to sound or socioeconomic background variables that might influence acoustic perception and wellbeing outcomes.

Further, context-specific variations have emerged across different educational settings. Urban settings consistently demonstrate more acoustic disturbances (Papanikolaou et al., 2013), whereas institutional differences between state funded and privately funded schools appear to influence noise perception patterns (Bulunuz, 2014). A noteworthy aspect emerged from the analysis of activity-specific variations within classrooms. Lundquist et al. (2003) demonstrated distinct noise patterns between classes of different subjects (e.g., language and mathematics, with language-based activities generating higher noise levels). Similarly, Astolfi et al. (2019) found higher average values of noise disturbance during quiet school activities than during group activities, suggesting that the nature of the teaching activity significantly influenced both the acoustic conditions and their perception. These findings highlight the importance of considering task-specific acoustic requirements in methodological design because different learning activities create distinct acoustic environments and subsequently impact wellbeing.

The results of this systematic review have substantial implications for educational policies and practice. This evidence strongly supports implementation of stringent acoustic standards in educational facilities. Specifically, the included studies emphasized three main aspects: the design of acoustically appropriate environments, development of acoustic awareness programmes, and the integration of acoustics as a fundamental element of educational wellbeing. Practical recommendations include the adoption of sound-absorbing systems, such as ceiling tiles or wall panels, implementation of noise reduction strategies, including reducing group size, rearranging classroom layouts, or scheduling noisier activities at times of lower cognitive load, development of educational programmes to raise student and teacher awareness of acoustics and their effects, and the creation of evidence-based guidelines for the management of acoustics in educational activities. Such guidelines may be informed by existing standards, such as the *Acoustics of Schools: a Design Guide* (Institute of Acoustics Association of Noise Consultants, 2015). As suggested by Vettori et al. (2024), specific interventions should target the different developmental stages of children, taking into account their evolving sensitivity to acoustic environments. For younger children who demonstrate less metacognitive awareness of acoustic conditions, interventions should prioritize experience-based learning approaches. These could include participatory activities such as making sound-absorbing panels in the classroom or engaging in interactive experiments on sound propagation, which allow children to directly experience and understand acoustic principles through hands-on engagement. By contrast, interventions for older students may effectively incorporate more theoretical and awareness-based approaches, relying on their enhanced metacognitive abilities and greater capacity to understand abstract acoustic concepts. These interventions should be considered fundamental components in the planning of educational facilities rather than optional improvements.

This review synthesizes evidence across educational levels, offering a comprehensive understanding of how classroom acoustics influences student experiences and wellbeing. However, some limitations of the included studies must be acknowledged. A notable geographical bias emerges, with approximately one-third of the studies conducted in Italy, potentially limiting the generalizability of the findings across different cultural and educational contexts. Future research should benefit from broader geographical representations to enhance cross-cultural understanding and validate the findings across diverse educational systems. Sample sizes varied substantially across studies (ranging from 50 to 2,036 participants), and the methodological approaches encompassed both quantitative and qualitative paradigms. This diversity, while offering rich insights into the phenomenon, introduces complexities into the comparability of the results. The methodological rigor of the acoustic measurements varied significantly across studies, and future research would benefit from more standardized measurement protocols, particularly in quantifying both objective acoustic parameters and the subjective experiences of students, ensuring more consistent and comparable research outcomes. The predominance of cross-sectional designs limits our understanding of long-term acoustic impacts; to address this limitation, longitudinal studies are needed to examine the sustained effects of acoustic conditions on educational outcomes and student wellbeing over time, providing insights into developmental trajectories and long-term implications. The relative underrepresentation of preschool and secondary school students and the limited investigation of specific populations, particularly students with special educational needs, in the reviewed literature limit the generalisability of the findings. Future research should prioritize these under-studied populations.

## 7 Conclusion

In conclusion, this systematic review demonstrated that acoustic quality is a determining factor in the wellbeing and educational experiences of students across all age groups. The evidence reveals a clear developmental trajectory in acoustic perception and sensitivity from younger students, who can accurately perceive noise conditions despite the limited conceptual understanding, to older students, who demonstrate greater acoustic sensitivity and awareness. This developmental model emphasizes the need for age-appropriate acoustic interventions and design solutions throughout the educational process. Poor acoustic conditions have emerged as a persistent challenge at all educational levels, with implications that go beyond immediate learning experiences and affect students' social relationships, emotional wellbeing, and overall engagement with the educational environment. While the physical features of classrooms can be objectively measured, their impact is meaningfully mediated by students' experiences, which reflect complex interactions between environmental conditions and psychological processes. The agreement between the students' subjective perceptions and objective acoustic measurements validated the importance of incorporating student feedback into acoustic assessments and improvement initiatives. By integrating acoustic modifications with educational interventions, schools can promote environments that foster better learning, communication, and engagement among students of all ages.

## Data Availability

The original contributions presented in the study are included in the article/Supplementary material, further inquiries can be directed to the corresponding author.

## Footnotes

^1^*Grundskola* is a Swedish compulsory school divided into nine levels from seven to sixteen years (https://grundskola.stockholm/en/).

## References

[B1] Astolfi A. Puglisi G. E. Murgia S. Minelli G. Pellerey F. Prato A. . (2019). Influence of classroom acoustics on noise disturbance and well-being for first graders. Front. Psychol. 10:2736. 10.3389/fpsyg.2019.02736 31920797 PMC6923245

[B2] Bablekou Z. Chrysochoou E. Kazi S. (2023). Executive functions, listening comprehension, and metacognitive processes in childhood: developmental profiles. Psychology 28, 48–68. 10.12681/psy_hps.36222

[B3] Battagliarin L. Spicciarelli G. Gheller F. Cappelletti F. Di Bella A. Paccagnella O. . (2024). “On the effect of indoor acoustic conditions in performing cognitive tests in primary school,” in INTER-NOISE and NOISE-CON Congress and Conference Proceedings, Vol. 270 (Institute of Noise Control Engineering), 8993–8999. 10.3397/IN_2024_4168

[B4] Bigozzi L. Di Cosimo A. Vettori G. (2016). Appearances are deceiving: observing the world as it looks and how it really is—theory of mind performances investigated in 3-, 4-, and 5-year-old children. Child Dev. Res. 2016:5270924. 10.1155/2016/5270924

[B5] Bradley J. S. (1986). Speech intelligibility studies in classrooms. J. Acoust. Soc. Am. 80, 846–854. 10.1121/1.393908 3760338

[B6] Brännström K. J. Johansson E. Vigertsson D. Morris D. J. Sahlén B. Lyberg-Åhlander V. (2017). How children perceive the acoustic environment of their school. Noise Health 19, 84–94. 10.4103/nah.NAH_33_16 29192618 PMC5437757

[B7] Bulunuz N. (2014). Noise pollution in Turkish elementary schools: evaluation of noise pollution awareness and sensitivity training. Int. J. Environ. Sci. Educ. 9, 215–234. 10.12973/ijese.2014.212a

[B8] Clark C. Paunovic K. (2018). WHO environmental noise guidelines for the European region: a systematic review on environmental noise and quality of life, wellbeing and mental health. Int. J. Environ. Res. Public Health 15:2400. 10.3390/ijerph15112400 30380665 PMC6266190

[B9] Connolly D. Dockrell J. Shield B. Conetta R. Mydlarz C. Cox T. (2019). The effects of classroom noise on the reading comprehension of adolescents. J. Acoust. Soc. Am. 145, 372–381. 10.1121/1.5087126 30710912

[B10] Connolly D. M. Dockrell J. E. Shield B. M. Conetta R. Cox T. J. Trevor J. (2013). Adolescents' perceptions of their school's acoustic environment: the development of an evidence based questionnaire. Noise Health 15, 269–280. 10.4103/1463-1741.113525 23771426

[B11] Dockrell J. E. Shield B. (2004). Children's perceptions of their acoustic environment at school and at home. J. Acoust. Soc. Am. 115, 2964–2973. 10.1121/1.1652610 15237821

[B12] Dockrell J. E. Shield B. (2012). The impact of sound-field systems on learning and attention in elementary school classrooms. J. Speech Lang. Hear. Res. 55, 1163–1176. 10.1044/1092-4388(2011/11-0026) 22232398

[B13] Dockrell J. E. Shield B. M. (2006). Acoustical barriers in classrooms: the impact of noise on performance in the classroom. Br. Educ. Res. J. 32, 509–525. 10.1080/01411920600635494

[B14] European Environment Agency X. X. (2010). Good Practice Guide on Noise Exposure and Potential Health Effects (Technical Report No. 11/2010). Available online at: https://www.eea.europa.eu/publications/good-practice-guide-on-noise (Accessed July 10, 2025).

[B15] Fernandes R. A. Vidor D. Oliveira A. A. (2019). The effect of noise on attention and performance in reading and writing tasks. CoDAS 31:e20170241. 10.1590/2317-1782/20182017241 31483038

[B16] Fernández-Quezada D. Martínez-Fernández D. E. Fuentes I. García-Estrada J. Luquin S. (2025). The influence of noise exposure on cognitive function in children and adolescents: a meta-analysis. NeuroSci 6:22. 10.3390/neurosci6010022 40137867 PMC11944768

[B17] Fretes G. Palau R. (2025). The impact of noise on learning in children and adolescents: a meta-analysis. Appl. Sci. 15:4128. 10.3390/app1508412839921717

[B18] Gathercole S. E. Pickering S. J. Knight C. Stegmann Z. (2004). Working memory skills and educational attainment: evidence from national curriculum assessments at 7 and 14 years of age. Appl. Cogn. Psychol. 18, 1–16. 10.1002/acp.934

[B19] Gheller F. Spicciarelli G. Scimemi P. Arfé B. (2024). The effects of noise on children's cognitive performance: a systematic review. Environ. Behav. 55, 698–734. 10.1177/00139165241245823

[B20] Gonzales C. (2015). The Role of Introspection in Children's Developing Theory of Mind. Arizona State University.

[B21] Gopnik A. Slaughter V. (1991). Young children's understanding of changes in their mental states. Child Dev. 62, 98–110. 10.2307/1130707

[B22] Hjetland H. N. Brinchmann E. I. Scherer R. Melby-Lervåg M. (2017). Preschool predictors of later reading comprehension ability: a systematic review. Campbell Syst. Rev. 13, 1–155. 10.4073/csr.2017.1437520794

[B23] Institute of Acoustics and Association of Noise Consultants (2015). Acoustics of Schools: A Design Guide. Institute of Acoustics. Available online at: https://www.ioa.org.uk/sites/default/files/Acoustics%20of%20Schools%20-%20a%20design%20guide%20November%202015_1.pdf (Accessed July 10, 2025).

[B24] International Organization for Standardization X. X.. (2008). Acoustics: Measurement of Room Acoustic Parameters. Reverberation Time in Ordinary Rooms. Durée de Réverbération Des Salles Ordinaires. International Organization for Standardization. Available online at: www.iso.org

[B25] Johnson S. P. Hannon E. E. (2015). “Perceptual development,” in Handbook of Child Psychology and Developmental Science: Vol. 2. Cognitive Processes, 7th Edn., eds. Liben L. S. Müller U. (Wiley), 63–112.

[B26] Klatte M. Bergström K. Lachmann T. (2013). Does noise affect learning? A short review on noise effects on cognitive performance in children. Front. Psychol. 4:578. 10.3389/fpsyg.2013.00578 24009598 PMC3757288

[B27] Klatte M. Hellbrück J. Seidel J. Leistner P. (2010a). Effects of classroom acoustics on performance and well-being in elementary school children: a field study. Environ. Behav. 42, 659–692. 10.1177/0013916509336813

[B28] Klatte M. Lachmann T. Meis M. (2010b). Effects of noise and reverberation on speech perception and listening comprehension of children and adults in a classroom-like setting. Noise Health 12, 270–282. 10.4103/1463-1741.70506 20871182

[B29] Kuhn D. (2000). Metacognitive development. Curr. Dir. Psychol. Sci. 9, 178–181. 10.1111/1467-8721.00088

[B30] Louca-Papaleontiou E. Melhuish E. Philaretou A. (2012). Introspective abilities of preschool children. Asian Trans. Basic Appl. Sci. 2, 14–30.

[B31] Lundquist P. Holmberg K. Burström L. Landström U. (2003). Sound levels in classrooms and effects on self-reported mood among school children. Percept. Mot. Skills 96, 1289–1299. 10.2466/pms.2003.96.3c.1289 12929784

[B32] Massonnié J. (2020). Understanding the Impact of Classroom Noise on Children's Learning and Well-being, and Its Modulation by Executive Functions (Doctoral Dissertation). Birkbeck, University of London.

[B33] Massonnié J. Frasseto P. Mareschal D. Kirkham N. Z. (2022). Learning in noisy classrooms: children's reports of annoyance and distraction from noise are associated with individual differences in mind-wandering and switching skills. Environ. Behav. 54, 58–88. 10.1177/0013916520950277

[B34] McFarland L. Dealtry L. (2017). Hearing in the early childhood setting: children's perspectives. Austral. J. Early Childh. 42, 105–113. 10.23965/AJEC.42.2.13

[B35] Mealings K. (2022). Classroom acoustics and cognition: a review of the effects of noise and reverberation on primary school children's attention and memory. Build. Acoust. 29, 401–431. 10.1177/1351010X221104892

[B36] Mealings K. Buchholz J. M. (2024). The effect of classroom acoustics and noise on high school students' listening, learning and well-being: a scoping review. Facilities 42, 485–503. 10.1108/F-06-2023-0049

[B37] Mogas-Recalde J. Palau R. Márquez M. (2021). How classroom acoustics influence students and teachers: a systematic literature review. J. Technol. Sci. Educ. 11, 245–259. 10.3926/jotse.109832969550

[B38] Ouzzani M. Hammady H. Fedorowicz Z. Elmagarmid A. (2016). Rayyan — a web and mobile app for systematic reviews. Syst. Rev. 5:210. 10.1186/s13643-016-0384-4 27919275 PMC5139140

[B39] Page M. J. McKenzie J. E. Bossuyt P. M. Boutron I. Hoffmann T. C. Mulrow C. D. . (2021). Updating guidance for reporting systematic reviews: development of the PRISMA 2020 statement. J. Clin. Epidemiol. 134, 103–112. 10.1016/j.jclinepi.2021.02.003 33577987

[B40] Papanikolaou M. Roussi C. Skenteris N. Katsioulis A. Piperakis S. M. (2013). Noise and perceived discomfort in Greek school children. J. Sci. Educ. 14, 40–42.

[B41] Papanikolaou M. Skenteris N. Piperakis S. M. (2015). Effect of external classroom noise on schoolchildren's reading and mathematics performance: correlation of noise levels and gender. Int. J. Adolesc. Med. Health 27, 25–29. 10.1515/ijamh-2014-0006 24810556

[B42] Pellegatti M. Torresin S. Visentin C. Babich F. Prodi N. (2023). Indoor soundscape, speech perception, and cognition in classrooms: a systematic review on the effects of ventilation-related sounds on students. Build. Environ. 236:110194. 10.1016/j.buildenv.2023.110194

[B43] Peng J. Wang D. Lau S. K. Yan N. Jiang P. Wu S. (2015). An investigation of acoustic treatment for children in a classroom of an elementary school. Appl. Acoust. 89, 42–45. 10.1016/j.apacoust.2014.09.005

[B44] Pirilä S. Jokitulppo J. Niemitalo-Haapola E. Yliherva A. Rantala L. (2020). Teachers' and children's experiences after an acoustic intervention and a noise-controlling workshop in two elementary classrooms. Folia Phoniatrica Logopaedica 72, 454–463. 10.1159/000503231 31639814

[B45] Shield B. M. Dockrell J. E. (2003). The effects of noise on children at school: a review. Build. Acoust. 10, 97–116. 10.1260/13510100376896596034902257

[B46] Skarlatos D. Manatakis M. (2003). Effects of classroom noise on students and teachers in Greece. Percept. Mot. Skills 96, 539–544. 10.2466/pms.2003.96.2.539 12776837

[B47] Stevens C. Bavelier D. (2012). The role of selective attention on academic foundations: a cognitive neuroscience perspective. Dev. Cogn. Neurosci. 2, S30–S48. 10.1016/j.dcn.2011.11.001 22682909 PMC3375497

[B48] Vettori G. Di Leonardo L. Secchi S. Astolfi A. Bigozzi L. (2022). Primary school children's verbal working memory performances in classrooms with different acoustic conditions. Cogn. Dev. 64:101256. 10.1016/j.cogdev.2022.10125640491944

[B49] Vettori G. Di Leonardo L. Secchi S. Bigozzi L. (2024). The sound of silence: Children's own perspectives on their hearing and listening in classrooms with different acoustic conditions. Eur. J. Psychol. Educ. 39, 3803–3823. 10.1007/s10212-024-00819-4

[B50] Visentin C. Torresin S. Pellegatti M. Prodi N. (2023). Indoor soundscape in primary school classrooms. J. Acoust. Soc. Am. 154, 1813–1826. 10.1121/10.0020833 37728288

[B51] Vrolijk M. Cebotari V. Richardson D. Cunsolo S. (2021). How Inquiring Develops and Affects Well-Being Throughout Childhood, Innocenti Working Paper 2021-14. Florence: UNICEF Office of Research – Innocenti.

[B52] Zelazo P. D. Craik F. I. Booth L. (2004). Executive function across the life span. Acta Psychol. 115, 167–183. 10.1016/j.actpsy.2003.12.005 14962399

