# Supplementary material for: The effects of classroom acoustic quality on student perception and wellbeing: a systematic review across educational levels
Source: Front Psychol. 2025 Aug 11;16:1586997. doi: 10.3389/fpsyg.2025.1586997 (PMC12375617; doi:10.3389/fpsyg.2025.1586997)
Supplement: Supplementary file 1 [file Supplementary_file_1.docx]

**eAppendix A. Keywords used in the search with EMBASE.**

| **Database: EMBASE** |
| --- |
| 1. 1S1 (child* OR children* OR childhood OR ‘early childhood’ OR infancy OR toddler* OR student* OR ‘early education’ OR preschool* OR kindergarten* OR grade* OR ‘primary school’ OR ‘school age’ OR ‘elementary school’ OR ‘formal education’ OR ‘pre-adolescent*’ OR ‘adolescent*’ OR ‘secondary school’) 2. S2 (class OR classroom OR ‘school classroom environment’ OR ‘educational setting*’ OR ‘educational environment*’ OR ‘learning environment’ OR ‘school*’ OR ‘environment*’ OR ‘school context*’ OR ‘academic institution*’) 3. S3 (noise* OR reverb* OR reverberation OR reverberance* OR resonance* OR sound* OR echo* OR ‘noise condition*’ OR ‘acoustic*’ OR ‘reverberation time*’ OR ‘babble noise*’ OR ‘road traffic noise’ OR ‘sound level*’ OR ‘noise exposure*’ OR ‘noisy educational setting*’ OR ‘environmental effect*’ OR ‘hear’ OR ‘hearing’) 4. S4 (silence* OR quiet OR quietness OR ‘quiet condition’ OR tranquility OR stillness OR calm* OR ‘acoustic quiet’ OR soundlessness OR ‘silence environment’ OR ‘noise-free environment’ OR ‘silence condition’ OR ‘absence of sound*’ OR ‘low-noise environment’ OR ‘acoustic calmness’) 5. S5 (‘metacognition*’ OR ‘metacognitive awareness’ OR ‘awareness’ OR ‘metacognitive understanding*’ OR ‘reflective cognition’ OR ‘sensory perception’ OR ‘sensory awareness’ OR ‘cognitive perception’ OR ‘cognitive awareness’ OR ‘environmental perception’ OR ‘environmental awareness’ OR ‘acoustic perception’ OR ‘silence perception’ OR ‘perspective’ OR ‘attitude*’) 6. S1 AND S2 AND S3 OR S4 AND S5 (1,151) |

Filters: Publication type, (Article, Article in press)

**Appendix B. Keywords used in the search with SCOPUS.**

| **Database: SCOPUS** |
| --- |
| 1. S1 (child* OR children* OR childhood OR ‘early childhood’ OR infancy OR toddler* OR student* OR ‘early education’ OR preschool* OR kindergarten* OR grade* OR ‘primary school’ OR ‘school age’ OR ‘elementary school’ OR ‘formal education’ OR ‘pre-adolescent*’ OR ‘adolescent*’ OR ‘secondary school’) 2. S2 (class OR classroom OR ‘school classroom environment’ OR ‘educational setting*’ OR ‘educational environment*’ OR ‘learning environment’ OR ‘school*’ OR ‘environment*’ OR ‘school context*’ OR ‘academic institution*’) 3. S3 (noise* OR reverb* OR reverberation OR reverberance* OR resonance* OR sound* OR echo* OR ‘noise condition*’ OR ‘acoustic*’ OR ‘reverberation time*’ OR ‘babble noise*’ OR ‘road traffic noise’ OR ‘sound level*’ OR ‘noise exposure*’ OR ‘noisy educational setting*’ OR ‘environmental effect*’ OR ‘hear’ OR ‘hearing’) 4. S4 (silence* OR quiet OR quietness OR ‘quiet condition’ OR tranquility OR stillness OR calm* OR ‘acoustic quiet’ OR soundlessness OR ‘silence environment’ OR ‘noise-free environment’ OR ‘silence condition’ OR ‘absence of sound*’ OR ‘low-noise environment’ OR ‘acoustic calmness’) 5. S5 (‘metacognition*’ OR ‘metacognitive awareness’ OR ‘awareness’ OR ‘metacognitive understanding*’ OR ‘reflective cognition’ OR ‘sensory perception’ OR ‘sensory awareness’ OR ‘cognitive perception’ OR ‘cognitive awareness’ OR ‘environmental perception’ OR ‘environmental awareness’ OR ‘acoustic perception’ OR ‘silence perception’ OR ‘perspective’ OR ‘attitude*’) 6. S1 AND S2 AND S3 OR S4 AND S5 (2,832) |

Filters: Publication type, (Article, Article in press)

**Appendix C. Keywords used in the search with PsycINFO and Education Source (via EbscoHost)**

| **Database: PsycINFO and Education Source** |
| --- |
| 1. S1 (child* OR children* OR childhood OR ‘early childhood’ OR infancy OR toddler* OR student* OR ‘early education’ OR preschool* OR kindergarten* OR grade* OR ‘primary school’ OR ‘school age’ OR ‘elementary school’ OR ‘formal education’ OR ‘pre-adolescent*’ OR ‘adolescent*’ OR ‘secondary school’) 2. S2 (class OR classroom OR ‘school classroom environment’ OR ‘educational setting*’ OR ‘educational environment*’ OR ‘learning environment’ OR ‘school*’ OR ‘environment*’ OR ‘school context*’ OR ‘academic institution*’) 3. S3 (nois* OR reverb* OR reverberation OR reverberance* OR resonance* OR sound* OR echo* OR ‘noise condition*’ OR ‘acoustic*’ OR ‘reverberation time*’ OR ‘babble noise*’ OR ‘road traffic noise’ OR ‘sound level*’ OR ‘noise exposure*’ OR ‘noisy educational setting*’ OR ‘environmental effect*’ OR ‘hear’ OR ‘hearing’) 4. S4 (silence* OR quiet OR quietness OR ‘quiet condition’ OR tranquility OR stillness OR calm* OR ‘acoustic quiet’ OR soundlessness OR ‘silence environment’ OR ‘noise-free environment’ OR ‘silence condition’ OR ‘absence of sound*’ OR ‘low-noise environment’ OR ‘acoustic calmness’) 5. S5 (‘metacognition*’ OR ‘metacognitive awareness’ OR ‘awareness’ OR ‘metacognitive understanding*’ OR ‘reflective cognition’ OR ‘sensory perception’ OR ‘sensory awareness’ OR ‘cognitive perception’ OR ‘cognitive awareness’ OR ‘environmental perception’ OR ‘environmental awareness’ OR ‘acoustic perception’ OR ‘silence perception’ OR ‘perspective’ OR ‘attitude*’) 6. S1 AND S2 AND S3 OR S4 AND S5 (862) |

Filters: Publication type, (Article, Article in press)

**Appendix D. Quality scores (risk of bias) of the studies according to the Quality Assessment Scale (Hjetland et al., 2017).**

| **Studies (in alphabetical order)** | **Sampling** | **Selection** | **Instrument quality** | **Test reliability** | **Floor or ceiling effect** | **Attrition** | **Missing data** | **Latent variables** | **Statistical power/sample size** | **Total score** |
| --- | --- | --- | --- | --- | --- | --- | --- | --- | --- | --- |
| **Astolfi et al. (2019)** | 1 | 1 | 1 | 1 | 1 | 0 | 1 | 0 | 0 | 6 |
| **Bulunz (2014)** | 1 | 0 | 1 | 0 | 1 | 1 | 1 | 1 | 0 | 6 |
| **Dockrell and Shield (2004)** | 0 | 0 | 1 | 0 | 1 | 0 | 0 | 1 | 0 | 3 |
| **Klatte et al. (2010)** | 1 | 1 | 0 | 0 | 1 | 0 | 0 | 0 | 0 | 3 |
| **Lundquist et al. (2003)** | 1 | 1 | 0 | 0 | 1 | 0 | 0 | 0 | 0 | 3 |
| **Massonnié et al. (2022)** | 1 | 1 | 1 | 0 | 1 | 0 | 0 | 0 | 1 | 5 |
| **McFarland and Dealtry (2017)** | 1 | 0 | 2 | 1 | 1 | 0 | 0 | 1 | 2 | 9 |
| **Papanikolaou et al. (2013)** | 0 | 0 | 2 | 1 | 1 | 0 | 0 | 1 | 0 | 5 |
| **Pirilä et al. (2020)** | 1 | 0 | 0 | 0 | 1 | 0 | 0 | 0 | 2 | 4 |
| **Skarlatos and Manatakis (2003)** | 1 | 0 | 1 | 2 | 1 | 1 | 1 | 1 | 0 | 8 |
| **Vettori et al. (2024)** | 1 | 0 | 1 | 0 | 1 | 0 | 0 | 0 | 0 | 3 |
| **Visentin et al. (2023)** | 1 | 1 | 0 | 0 | 1 | 0 | 0 | 0 | 1 | 4 |

| **Appendix E. A synthesis of the characteristics, method and results of the studies included.** | | | | | | | |
| --- | --- | --- | --- | --- | --- | --- | --- |
| **Studies (in alphabetical order)** | **Methodological approach** | **Country** | **Sample (n)** | **Educational level** | **Age range or school grade** | **Measures** | **Results** |
| Astolfi et al. (2019) | Quantitative | Italy | 340 children | Primary school | 6–8 y/o | Acoustic measurements reverberation time (ISO 3382-2, 2008) and speech clarity (ISO 3382-1, 2009).  Questionnaire for perceived well-being assessment - Italian translation (Sabri et al., 2015).  Questionnaire on noise disturbance (adapted from Dockrell & Shield, 2004; Astolfi & Pellerey, 2008). | Long reverberation time in the classroom leads to a lower perception of both enjoyment and happiness among students. Increased perception of noise disturbance between happy children in classrooms with poor acoustics.  The noise in classrooms with higher reverberation, either from outside or inside, can be perceived as more disturbing as it is amplified more by reverberation.  Seven out of 12 well-being items were rated significantly worse by unhappy students in good acoustics, compared to 11 out of 12 in bad acoustics. To 11 out of 12 in poor acoustics, suggesting that poor classroom acoustics are less determinant of well-being than good acoustics. |
| Bulunz (2014) | Mixed-method | Turkey | 611 children (263 + 348)  48 teachers (22 + 26) | Primary school | 7–13 y/o | Noise levels (Svan 957 sound level metre and analyser).  Noise Pollution Questionnaire (ad-hoc questionnaire) | Students could perceive and report noise levels in their educational environment. The agreement between students’ subjective perceptions and the objective reverberation measurements confirms the children’s ability to perceive acoustic conditions, despite institutional differences (state funded and privately funded school). Moreover, 69.3% of the students in the privately funded school stated that noise pollution was present in their school, and 73.1% were annoyed by it. These are lower values compared to the 83.9% and 83% of the students in state funded schools, respectively. |
| Dockrell and Shield (2004) | Quantitative | United Kingdom | 2036 children | Primary school | 6–7 y/o and 10–11 y/o | Questionnaire on sound sources children were exposed to in  their environment.  Smiley faces rating scale (based Arnold & Canning, 1999) | Children’s reported ability to hear the teacher varied significantly across situations (χ^2^ = 4426, p < .001), with ‘no noise outside the classroom’ and ‘doing a test’ reported as the best listening conditions and ‘noise from other children outside’ the worst. Younger children generally reported that it was significantly more difficult to hear the teacher.  There was a significant difference between the two age groups in six of the nine situations assessed: when the teacher was ‘talking and moving’ (U = 394 579.5, p < .001); ‘no noise outside’ (U = 394 382, p < . 001); ‘doing a test’ (U = 355 254.5, p < .001); ‘PE in the playground’ (U = 461 915.5, p < .001); ‘no noise at all’ (U = 409 882.5, p < .001); and ‘classmate speaking’ (U = 418 452, p < .05). Compared to the younger children, the older children reported significantly greater difficulties when they could not see the teacher’s face (U = 392 595.5, p < .001) and when ‘children were making noise outside the classroom’ (U = 423 164, p < .001).  There were no group differences in reported hearing acuity when children worked in groups (U = 490 863.5, ns).  These results suggest that primary school children can judge situations in which they have difficulty hearing the teacher and that younger children report relatively greater difficulty than older children. Thus, children can discriminate between situations with different levels and types of noise. |
| Klatte et al. (2010) | Quantitative | Germany | 487 children | Primary school | 1–2 grade ,  7 - 8 y/o | Acoustic measurements reverberation time.  Salzburger Lesescreening (SLS;  Mayringer & Wimmer, 2003)  Hamburger Schreibprobe (HSP; May, 2002)  Coloured Progressive Matrices (Raven, 2002; Nicklos, 1967),  Assessment of phonological  processing, noise questionnaire (Henze, 2006),  Questionnaire on Emotional and Social School Experiences of Elementary School Children, (FEESS; Rauer & Schuck, 2004) | Significant effect of classroom reverberation time on indoor noise annoyance, F(2, 372) = 11.81, p < .001. Indoor noise ratings were lower for children whose classrooms had short reverberation times compared to children from classrooms with medium and long reverberation times (p < .001 in both cases).  One-way analyses of variance (ANOVA) show significant effects of reverberation were found for the scales achievement motivation, relationship with teachers, classroom climate and social integration (p < .01 in all cases). Bonferroni-corrected post-hoc tests showed that children from classrooms with long reverberation (RT_3) rated these aspects less positively than children from classrooms with medium (RT_2) and short reverberation (RT_l), p < .05 in both cases. |
| Lundquist et al. (2003) | Quantitative | Sweden | 443 children | Secondary school | 12–15 y/o | Sound level metre  and a digital tape recorder.  Mood questionnaire (adjective check-list on Task Orientation and Inattentiveness). | During lessons, sound levels were caused by pupil activity, talking and furniture scraping. However, the research does not support the evidence of a correlation between activity sound levels and background sound levels or variation in the number of pupils in each class or year group (F =3.0, p > .05) and does not support the idea that higher sound levels should increase annoyance and inattention. |
| Massonnié et al. (2022) | Quantitative | France | 112 children | Primary school | 8–11 y/o | Children’s reactions to noise questionnaire  Attentional Control Switching Scale (Carriere et al., 2013),  Mind wandering questionnaire (Mrazek et al., 2013) | Factor analysis identified five key factors in children’s responses to noise: perceived noise level, attentional capture, reported hearing difficulties, interference and annoyance. Structural equation modelling confirmed that interference and annoyance were distinct but correlated constructs. More severe hearing difficulties increased both interference (β = .34, p = .01) and annoyance (β = .31, p = .02), with a marginal effect of interference on annoyance (β = .21, p = .06). Mind wandering increased interference (β = .63, p < .001) and indirectly led to greater annoyance (β = .18, p = .02). Finally, better switching skills reduced both interference (β = -.61, p < .001) and annoyance (β = -.60, p < .001). |
| McFarland and Dealtry (2017) | Qualitative | Australia | 69 children  32 parents  9 educators | Preschool | 3–5 y/o | Self-administered booklet on children’s perceptions of hearing (combination of verbal, affective and visual/arts-based expression) | Children reported the least difficulty with ‘listening to a story on the mat’ (M = 1.36, SD = 0.59), ‘listening to the smartboard on the mat’ (M = 1.44, SD = 0.71) and ‘sitting at the front of the mat’ (M = 1.24, SD = 0.47).  Conversely, the most challenging situations were ‘listening to a story on the mat when other children are talking’ (M = 2.25, SD = 0.69) and ‘sitting at the back of the mat’ (M = 1.81, SD = 0.82).  Based on their self-reports, 12 children were identified as having potential hearing difficulties as their scores were more than one standard deviation below the mean. |
| Papanikolaou et al. (2013) | Quantitative | Greece | 594 children | Primary school | 5–6 grade | Ad-hoc questionnaire for children to record the most common types of noise during lessons and the distress caused by noise. | Children perceive external noise mainly from traffic, construction and adult voices, while the most annoying internal noise comes from classmates and school corridors.  The ANOVA results indicate significant differences in the reported annoyance between the three cities. Regarding internal noise, children from Larissa were more bothered by corridor noise (F = 4.33, df = 2, p < 0.05), while children from Volos were less bothered by noise from neighbouring classrooms (F = 4.93, df = 2, p < .01). Further, a significant sex difference was found in the perception of the impact of noise on academic performance (F = 3.86, df = 1,590, p < 0.05), with girls reporting higher annoyance than boys. |
| Pirilä et al.(2020) | Quantitative | Finland | 50 children  2 teachers | Primary school | 8–9 y/o  11–12 y/o | Acoustic measurements reverberation time.  Questionnaire on teachers’ and pupils’ experiences of annoying noises in the classroom  Questionnaire on students’ experiences of the teacher’s voice | After the interventions, the younger and older students showed significant changes in their sound experiences (F(2, 96) = 5.718, p = .005). The older students reported a significant improvement in the clarity and audibility of the teacher’s voice (p = .005) and a reduction in the concentration required to hear the teacher (p = .016). Both groups reported a significant reduction in annoyance caused by noise from furniture and the corridor (p = .015). Group B reported a significant decrease in annoyance caused by other pupils talking (p = .005). |
| Skarlatos and Manatakis (2003) | Quantitative | Greece | 411 children  130 teachers | High school | 12–20 y/o | Acoustic measurements:  Noise level (B&K 2221 Precision Integrating Sound Level Meter and CEL 593 Sound Level Analyzer) and  reverberation time (dB4 Building Acoustics software)  Questionnaire on student and teacher perception of noise levels in the classroom | The noise level in the classroom depended on the time period and the number of students. Noise levels increased with the number of students and the time period but decreased with the average age of the students. The R² statistic was 0.32 (adjusted R² = 0.29), indicating a moderate explained variance of noise. The Pearson correlation analysed the relationship between classroom noise levels and students’ perceived discomfort. The results showed a positive relationship between student age and noise related discomfort (r = 0.61, p < .01). While the partial correlation controlling for age and time period was r = 0.31 (p = .02). |
| Vettori et al. (2024) | Quantitative | Italy | 213 children | Primary school | 6–11 y/o | Acoustic measurements reverberation time.  Questionnaire on children’s perspectives of listening during learning | Two-way ANOVA showed no significant main effect of acoustic condition (F(1,203) = 2.58, p = .110, η2 = 0.01) or school grade (F(4,203) = 1.00, p = .406, η2 = 0.02) on children’s perspectives of listening during learning.  There was a significant interaction effect between acoustic condition and grade, F(4,203) = 3.33, p < 0.05, η2 = 0.06.  Fourth-grade children in the good acoustic condition scored significantly higher (M = 25.52, SD = 3.37) than children in the poor acoustic condition (M = 23.41, SD = 3.43).  Fifth-grade children in the good acoustic condition scored significantly higher (M = 26.12, SD = 3.73) than children in the poor acoustic condition (M = 23.00, SD = 2.11).  There was a significant difference between the fourth-grade classes, t(41) = 2.04, p < 0.05, Cohen’s d = - 0.62,  and fifth-grade classes, t(46) = 3.52, p < .001, Cohen’s d = -1.02. |
| Visentin et al. (2023) | Quantitative | Italy | 130 children | Primary school | 8–10 y/o | Acoustic measurements: background noise level and reverberation time (T30) and clarity (C50)  Measured according to ISO 3382-2.  Questionnaire on children’s  subjective perception of the actual sound environment of their classroom and their ideal sound environment. | Students’ voices were the most common, followed by indoor and outdoor noise, most of which was perceived as unpleasant. Increased perceived noise significantly decreased comfort, while children’s voices in neighbouring classrooms correlated positively with comfort (17.2% explained variance). Younger pupils felt more comfortable than older pupils (median difference = 0.46, p = .009). Higher loudness and children’s voices increased perceived arousal (40.9% explained variance). Students preferred environments without anthropogenic sounds, with no significant relationship between actual noise perception and ideal soundscape preference. |
